# Commentary on Raghuraman et al. (2021). On the Long-Term Efficacy and Effectiveness of Narrative Exposure Therapy

**DOI:** 10.3389/fpsyg.2021.771958

**Published:** 2021-11-08

**Authors:** Sebastian Siehl, Laura Wilhelms, Anselm Crombach

**Affiliations:** ^1^Institute of Medical Psychology and Medical Sociology, University Medical Center Schleswig-Holstein, Kiel University, Kiel, Germany; ^2^Institute of Cognitive and Clinical Neuroscience, Central Institute of Mental Health, Medical Faculty Mannheim, Ruprecht-Karls-University Heidelberg, Mannheim, Germany; ^3^Non-governmental Organization vivo International e.V., Konstanz, Germany; ^4^Department of Psychology, Clinical Psychology and Psychotherapy for Children and Adolescents at the University of Saarland, Saarbrücken, Germany; ^5^Department of Psychology at the University of Konstanz, Clinical Psychology, Kosntanz, Germany; ^6^Non-governmental Organization Psychologues Sans Frontières, Bujumbura, Burundi; ^7^Department of Clinical Psychology, Université Lumière de Bujumbura, Bujumbura, Burundi

**Keywords:** commentary articles, PTSD–post-traumatic stress disorder, narrative exposure therapy (NET), systematic review and meta-analysis, long-term effects

In the past years, several systematic reviews and meta-analyses have been published assessing the effectiveness of narrative exposure therapy [NET; (Lely et al., 2019; Raghuraman et al., 2021; Siehl et al., 2021; Wei and Chen, 2021)]. The meta-analyses had different aims and came to different conclusions about the effectiveness of NET. Lely et al. (2019) and Wei and Chen (2021) focused on between-treatment effects, post intervention, comparing NET with active and non-active control-treatment-conditions. In addition to comparing between-treatment effects Raghuraman et al. (2021) and Siehl et al. (2021) assessed also the temporal stability of the effects. The latter two studies investigated the reduction of symptom severity of posttraumatic stress disorder (PTSD) and the percentage of PTSD diagnoses over several follow-up periods. Raghuraman et al. (2021) indicated a medium standardized mean difference (SMD) in favor of NET in comparison to active and inactive control groups in the long-term and no benefit regarding PTSD diagnoses. The authors cautioned against using the existing evidence to inform policies and guidelines. In contrast, Siehl et al. (2021) found a large SMD in favor of NET compared to active or inactive control groups in the long-term. They reported an improvement of effectiveness over time when analyzing active control groups and concluded that NET is an effective treatment approach in post-conflict settings and refugee populations, highlighting the high external validity of the trials. Acknowledging the significant efforts of both author groups to select, code, and analyze the existing evidence, we aim to clarify potential underlying reasons for the differences between the two meta-analyses. The purpose of this commentary is two-fold: (a) discuss more generally ways to assess the quality of a treatment, such as NET, that is used in a broad range of contexts and (b) more specific differences between the two meta-analyses in (1) selecting and analyzing strategies, and (2) potential coding errors.

A variety of tools and methods are used to ensure the quality of systematic reviews and meta-analyses (Higgins et al., 2011) to evaluate the effectiveness of psychotherapeutic interventions, such as NET, in comparison to control conditions. These include the preregistration on platforms like the *international prospective register of systematic reviews* (PROSPERO; https://www.crd.york.ac.uk/prospero/) or *Cochrane* (https://www.cochrane.org) to a priori define the research question, hypotheses, inclusion/exclusion criteria and planned types of analysis, quality control and the use of risk of bias assessments (RoB) like the Cochrane RoB tool (Higgins et al., 2011). Following the guidelines and using these tools limits systematic errors and offers a possibility to evaluate the primary studies qualitatively. Whereas, both meta-analyses (Raghuraman et al., 2021; Siehl et al., 2021) used the Cochrane RoB tool, Raghuraman et al. (2021) did not pre-register their meta-analysis. However, attempting to resolve quality control-related issues with checklists and preregistrations cannot explain the differences between the two meta-analyses. After intense discussions, the most plausible reasons seem differences in the selection and analysis process as well as potential coding errors.

One reason for the disparate results might be the different operationalizations of long-term. Raghuraman et al. (2021) defined “long-term” as 12 months and more. Siehl et al. (2021) defined “long-term” as 6 months and more. However, even the mid-term results (6–7 months) from Raghuraman et al. (2021) yield only a moderate effect in favor of NET regarding PTSD symptom severity. Hence, this explanation seems unlikely. Another reason might be the different selection criteria of the included studies. Raghuraman et al. (2021) included 24 studies using the following inclusion criteria: (a) individuals with a history of exposure to trauma and PTSD outcome measure, (b) randomized controlled study (RCT). While the authors set a restriction on type of therapy, not including NET for children (KIDNET), the authors did include studies with underage populations (Hermenau et al., 2013; Al-Hadethe et al., 2015). They further conducted an analysis combining NET for adults and adaptations for perpetrators (Forensic Offender Rehabilitation NET; FORNET). Siehl et al. (2021) separately coded NET, KIDNET, and FORNET studies. We argue in favor of separating these versions of NET when assessing their efficacy (Siehl et al., 2021) noting a potential age effect regarding the efficacy of NET (Lely et al., 2019), the possible impact of differences in brain maturity and in cognitive development on treatment efficacy (Lenroot and Giedd, 2006), particularities regarding beneficial effects for perpetrators, as well as subsequent adaptations of NET (Stenmark et al., 2014; Hecker et al., 2015). Doing so counteracts heterogeneity that arises when pooling data from different populations and forms of treatment.

Even though these aspects partially explain the different results, other aspects regarding methodology and coding possibly contribute significantly to the disparate conclusions. Aiming to replicate the analyses of Raghuraman et al. (2021) we recoded the included articles in line with the described procedures and compared the results to those reported in the original paper. It was unclear why the authors excluded most NET studies with children but included FORNET studies. Furthermore, by recoding the included studies we identified considerable outcome differences in 4 out of 6 studies regarding PTSD symptom severity, and in 1 out of 3 studies regarding the percentage of PTSD diagnoses (see Figure 1). Unfortunately, we could not replicate the results as it remained unclear which data was chosen and coded as the control condition. In our replication of the meta-analysis, we came to an overall SMD of 0.87 (*Z* = 4.05) in comparison to the results by Raghuraman et al. (2021) with an SMD of 0.49 (*Z* = 3.06). The following reasons may explain some of the differences: (1) Ertl et al. (2011): a coding error might have occurred; specifically, the interchanging of the percentage of participants no longer fulfilling the criteria for PTSD has possibly led to the opposing results regarding the diagnosis of PTSD (Ertl et al., 2011, p. 510); (2) In the cases of Hinsberger et al. (2020), Neuner et al. (2004), and Ertl et al. (2011) it remained unclear which data was chosen and coded as the control condition in the meta-analysis of symptom severity, because the results reported in these studies are incompatible with those in Raghuraman et al. (2021). We attempted to replicate the results of Raghuraman et al. (2021) with the data available in the original studies, we did not contact them for additional information. Furthermore, we would like to highlight that the reduction of symptoms in highly affected populations like survivors of genocide cannot be captured using binary categories of PTSD diagnosis.

**Figure 1:**
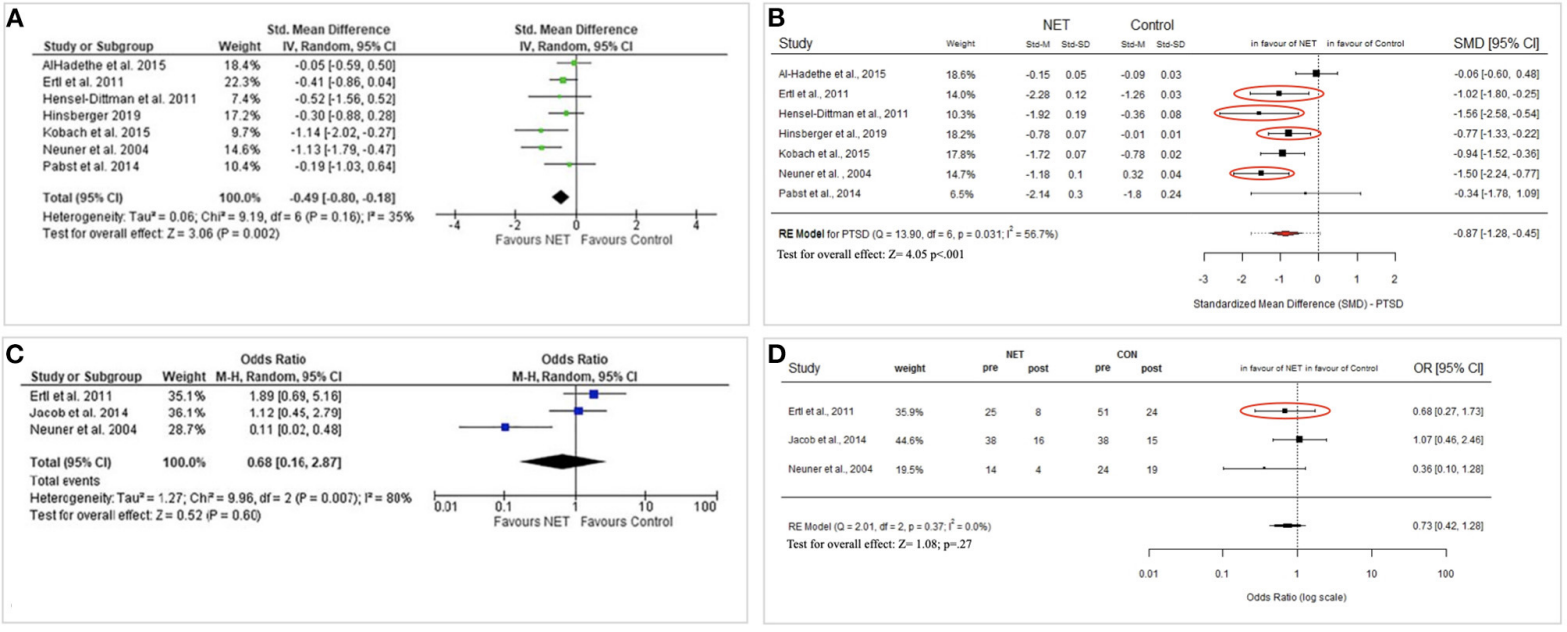
**(A)** Symptom severity of PTSD (longterm) taken from Raghuraman et al. (2021, Figure B10). **(B)** Reconstructed data for symptom severity of PTSD (longterm). **(C)** Diagnosis of PTSD (longterm) taken from Raghuraman et al. (2021, Figure B7). **(D)** Reconstructed data for diagnosis of PTSD (longterm). Red circles indicate differences regarding the reconstructed results.

In conclusion, both meta-analyses support the effectiveness of NET in comparison with other treatments and found a stable effect over time. However, we demonstrated that the results of meta-analyses are significantly affected by design, potential omissions during study inclusions, challenges during coding procedures, and unclear methodological descriptions, both in the source articles and meta-analyses. Safety procedures, including preregistration and double-checking results, must be implemented with great care. However, even though strict criteria, emphasized, for example, by Cochrane, are very important, we stress that meta-analyses often focus on clinical studies at Phase-III with efficacy studies focusing on RCTs. Here, the internal validity is highly important (Buchkremer and Klingberg, 2001). A large amount of NET studies can be considered to be Phase-IV studies, assessing the effectiveness of an intervention under more naturalistic settings, broadening the context (cultural contexts, community settings, types of populations) in which an intervention is applied with a focus on external validity. In the case of NET, a treatment for vulnerable populations, the latter seems particularly important. Hence, many NET studies have been conducted aiming to assess the external validity of NET in multiple cultural contexts, community settings, and across different populations. This strength, however, is rarely reflected in meta-analyses. We argue that there is evidence on the long-term efficacy of NET. Nevertheless, more classic RCT-Phase III and Phase-IV studies are needed in the case of NET, as is arguably the case for most trauma-focused treatments.

## Author Contributions

SS and LW organized the database. SS performed the statistical analysis. All authors contributed to conception and design of the study, wrote the first draft of the manuscript, and contributed to manuscript revision, read, and approved the submitted version.

## Conflict of Interest

The authors declare that the research was conducted in the absence of any commercial or financial relationships that could be construed as a potential conflict of interest.

## Publisher's Note

All claims expressed in this article are solely those of the authors and do not necessarily represent those of their affiliated organizations, or those of the publisher, the editors and the reviewers. Any product that may be evaluated in this article, or claim that may be made by its manufacturer, is not guaranteed or endorsed by the publisher.
